# Genetic Diversity of Polymyxin-Resistance Mechanisms in Clinical Isolates of Carbapenem-Resistant Klebsiella pneumoniae: a Multicenter Study in China

**DOI:** 10.1128/spectrum.05231-22

**Published:** 2023-02-27

**Authors:** Ziyao Li, Xinmeng Liu, Zichen Lei, Chen Li, Feilong Zhang, Yongli Wu, Xinrui Yang, Jiankang Zhao, Yulin Zhang, Yanning Hu, Fangfang Shen, Pingbang Wang, Junwen Yang, Yulei Liu, Binghuai Lu

**Affiliations:** a China-Japan Friendship Institute of Clinical Medical Sciences, Beijing, China; b Laboratory of Clinical Microbiology and Infectious Diseases, Department of Pulmonary and Critical Care Medicine, Center of Respiratory Medicine, National Clinical Research Center for Respiratory Diseases, National Center for Respiratory Medicine, China-Japan Friendship Hospital, Beijing, China; c Institute of Respiratory Medicine, Chinese Academy of Medical Sciences, Beijing, China; d Peking Union Medical College, Chinese Academy of Medical Sciences, Beijing, China; e Peking University China-Japan Friendship School of Clinical Medicine, Beijing, China; f Liuyang Traditional Chinese Medicine Hospital, Changsha, Hunan, China; g Heping Hospital affiliated with Changzhi Medical College, Changzhi, Shanxi, China; h The People’s Hospital of Liuyang, Changsha, Hunan, China; i Department of Laboratory Medicine, Zhengzhou Key Laboratory of Children’s Infection and Immunity, Children’s Hospital Affiliated with Zhengzhou University, Zhengzhou, Henan, China; j Department of Laboratory Medicine, Beijing Anzhen Hospital, Beijing, China; Brown University

**Keywords:** polymyxin resistance mechanism, carbapenemase-resistant *Klebsiella pneumoniae*, antibiotic resistance, *mgrB* inactivation, *K. quasipneumoniae*, genetic diversity, multicenter study, polymyxin-resistant *Klebsiella pneumoniae*

## Abstract

Polymyxin has been the last resort to treat multidrug-resistant Klebsiella pneumonia. However, recent studies have revealed that polymyxin-resistant carbapenem-resistant Klebsiella pneumonia (PR-CRKP) emerged due to the mutations in chromosomal genes or the plasmid-harboring *mcr* gene, leading to lipopolysaccharide modification or efflux of polymyxin through pumps. Further surveillance was required. In the present study we collected PR-CRKP strains from 8 hospitals in 6 provinces/cities across China to identify the carbapenemase and polymyxin resistance genes and epidemiological features by whole-genome sequencing (WGS). The broth microdilution method (BMD) was performed to determine the MIC of polymyxin. Of 662 nonduplicate CRKP strains, 15.26% (101/662) were defined as PR-CRKP; 10 (9.90%) were confirmed as Klebsiella quasipneumoniae by WGS. The strains were further classified into 21 individual sequence types (STs) by using multilocus sequence typing (MLST), with ST11 being prevalent (68/101, 67.33%). Five carbapenemase types were identified among 92 CR-PRKP, *bla*_KPC-2_ (66.67%), *bla*_NDM-1_ (16.83%), *bla*_NDM-5_ (0.99%), *bla*_IMP-4_ (4.95%), and *bla*_IMP-38_ (0.99%). Notably, 2 PR-CRKP strains harbored both *bla*_KPC-2_ and *bla*_NDM-1_. The inactivation of *mgrB*, associated significantly with high-level polymyxin resistance, was mainly caused by the insertion sequence (IS) insertion (62.96%, 17/27). Furthermore, *acrR* was inserted coincidently by IS*kpn26* (67/101, 66.33%). The deletion or splicing mutations of *crrCAB* were significantly associated with ST11 and KL47 (capsule locus types), and diverse mutations of the *ramR* gene were identified. Only one strain carried the *mcr* gene. In summary, the high IS-inserted *mgrB* inactivation, the close relationship between ST11 and the deletion or splicing mutations of the *crrCAB*, and the specific features of PR-*K. quasipneumoniae* constituted notable features of our PR-CRKP strains in China.

**IMPORTANCE** Polymyxin-resistant CRKP is a serious public health threat whose resistance mechanisms should be under continuous surveillance. Here, we collected 662 nonduplicate CRKP strains across China to identify the carbapenemase and polymyxin resistance genes and epidemiological features. Polymyxin resistance mechanism in 101 PR-CRKP strains in China were also investigated, 9.8% of which (10/101) were *K. quasipneumoniae*, as determined via WGS, and inactivation of *mgrB* remained the most crucial polymyxin resistance mechanism, significantly related to high-level resistance. Deletion or splicing mutations of *crrCAB* were significantly associated with ST11 and KL47. Diverse mutations of the *ramR* gene were identified. The plasmid complementation experiment and mRNA expression analysis further confirmed that the *mgrB* promoter and *ramR* played a critical role in polymyxin resistance. This multicenter study contributed to the understanding of antibiotic resistance forms in China.

## INTRODUCTION

Carbapenem-resistant Klebsiella pneumoniae (CRKP) was highlighted as a notorious pathogen of various infections (1). Polymyxin (polymyxin B and colistin) has been the last-resort treatment for such infections, even with an unfavorable side effect profile, including nephrotoxicity and neurotoxicity (2). However, the emergence of polymyxin resistance has been documented in the setting of increased use and imposed negative impacts on its usage in CRKP treatment (2, 3).

Polymyxin-resistant K. pneumoniae (PRKP) can be conferred by mutations in the two-component system (TCS), *mgrB*, efflux pumps, repressors, and lipopolysaccharide (LPS)-modifying genes (2). The upregulation of TCS genes, such as *phoP*/*phoQ* and *pmrA*/*pmrB*, also confers resistance to polymyxins. MgrB is a negative regulator of PhoQ. The insertion sequence (IS)-mediated *mgrB* gene disruption and inactivation in *mgrB* and its promoter region constituted the main polymyxin resistance mechanism in K. pneumoniae (4, 5). The addition of 4-amino-4-deoxy-l-arabinose (l-Ara4N) and/or phosphoethanolamine (pEtN) to the LPS was mediated by the *arnBCADTEF* and *pmrCAB* operons, respectively (2). PmrD, encoded by *pmrD*, is a connector protein that might activate PmrB/PmrA in response to PhoQ/PhoP stimulation (6). Furthermore, many other mechanisms are involved simultaneously in polymyxin resistance. For example, the *ramA*, *yciM*, and *lpxM* genes, which can mediate LPS alterations, along with the AcrAB-TolC pump and its regulators (*acrAB*, *kpnEF*, and *SoxSR*), contribute to mediating decreased susceptibility of polymyxin in K. pneumoniae (7–11). The plasmid-borne *mcr*-1 gene and its variant-mediated polymyxin resistance have also been reported in K. pneumoniae in the hospital setting (2, 12).

Moreover, Klebsiella quasipneumoniae was a newly designated species in Klebsiella spp. Few reports described its polymyxin- and carbapenem-resistance molecular features since it was not generally distinguished from K. pneumoniae by matrix-assisted laser desorption ionization–time of flight mass spectrometry (MALDI-TOF MS) and routine biochemical methods in the clinical laboratory (13).

The increasing rate in clinical isolates of polymyxin- and carbapenem-resistant K. pneumoniae (PR-CRKP) warrants further investigation into their epidemiology and underlying molecular mechanisms. Here, we conducted the study aiming to determine the prevalence of PR-CRKP in six provinces/cities in China, their clonal relationships, and the molecular features via whole-genome sequence (WGS) and bioinformatics analysis.

## RESULTS

### General characteristics of CRKP and PR-CRKP.

**FIG 1:**
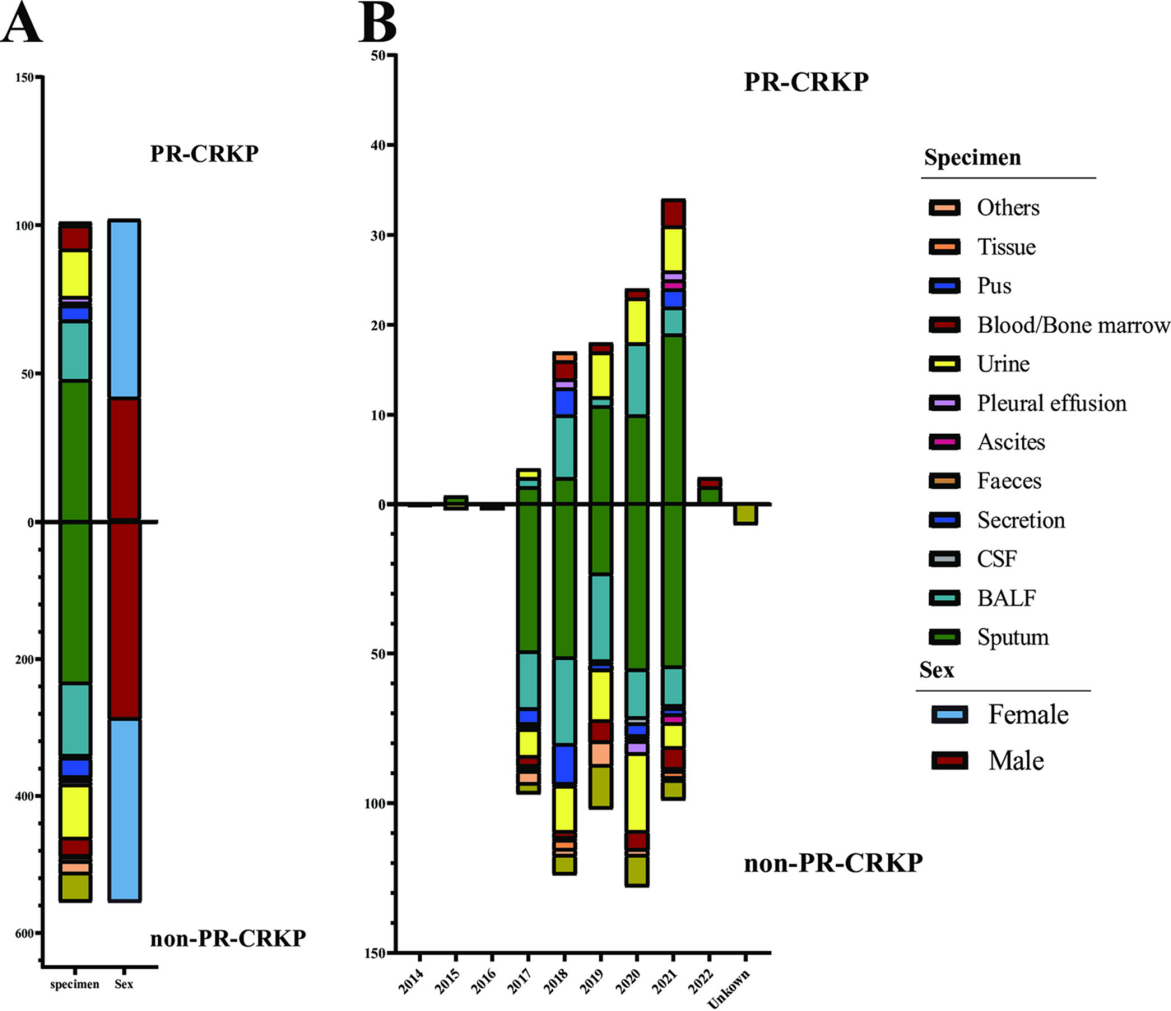
Sample source and distribution. (A) Sample distribution of 101 PR-CRKP strains and 561 non-PR-CRKP strains and the sex of the patients. (B) The number of strains isolated from different specimen types per year among PR-CRKP and non-PR-CRKP.

As detailed in Fig. 1, of 662 unduplicated CRKP strains, 15.26% (101/662) were classified as PR-CRKP; 411 (62.08%, 411/662) were recovered from the lower respiratory tract, followed by urine (14.20%, 94/662), secretion (4.83%, 32/662), and blood (4.68%, 31/662). The gender ratio of male/female was approximately 1:1 (333/329). The enrolled patients were aged from 1 day to 102 years, with a median age of 64 years.

**FIG 2:**
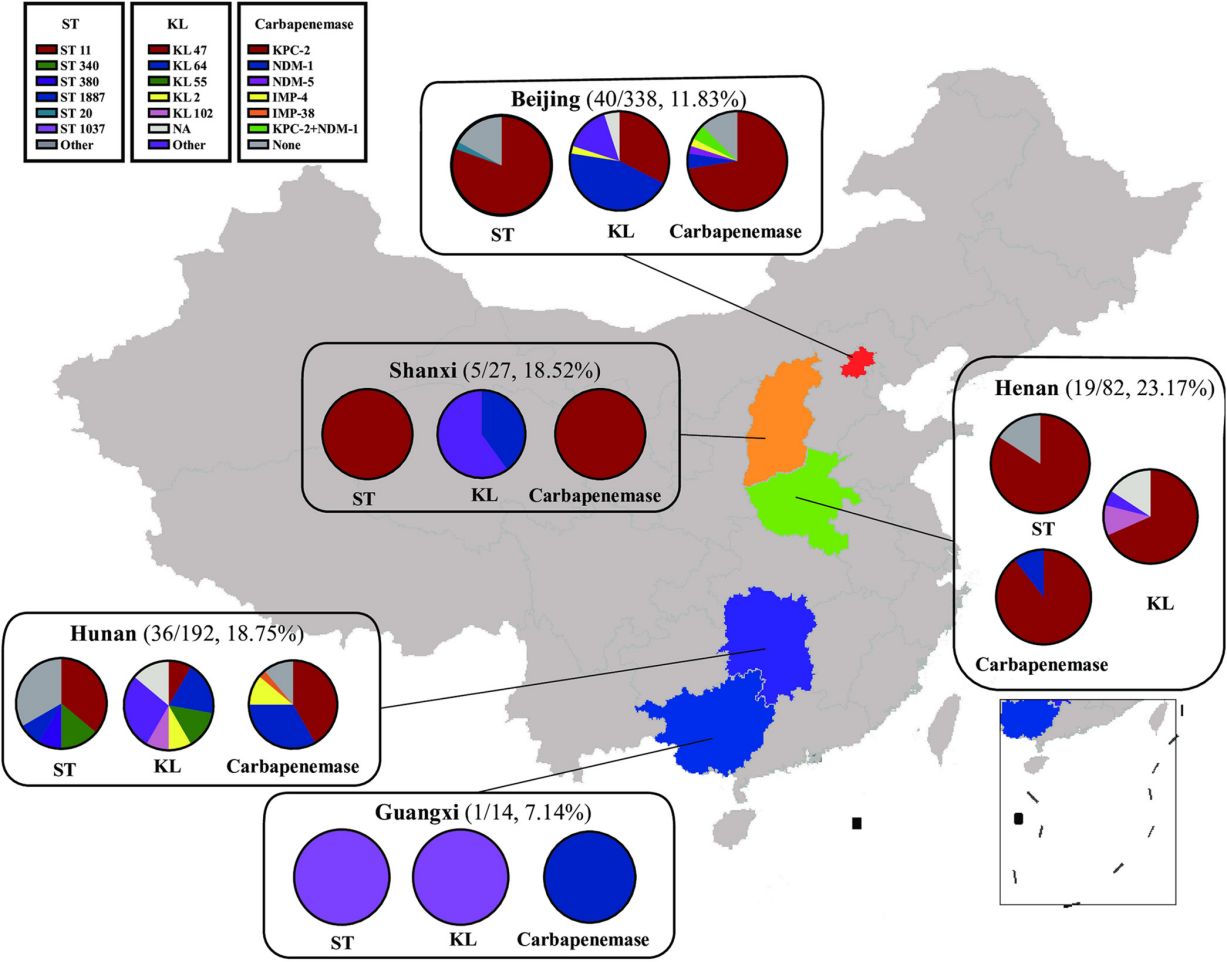
Geographical locations of polymyxin- and carbapenem-resistant K. pneumoniae (PR-CRKP) strains circulating in China. The color-highlighted provinces/cities are those where PR-CRKP strains were recovered, with the number of strains of PR-CRKP/CRKP shown in parentheses. The distribution of sequence types (ST), capsule locus types (KL types), and carbapenemase genes were also shown. Nine CRKP strains were detected in Hebei, while none were resistant to polymyxin.

**FIG 3:**
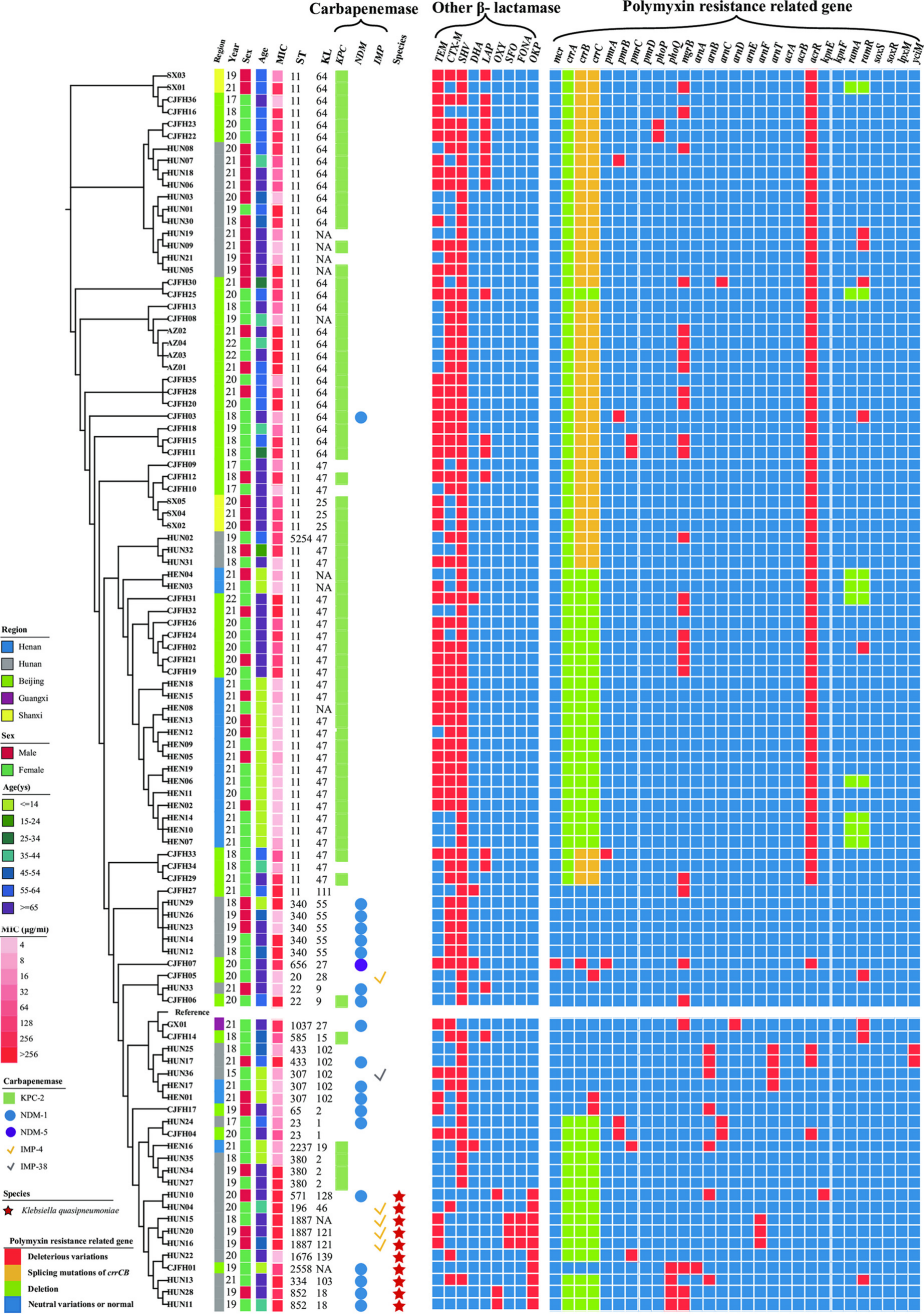
Phylogenetic tree diagram of 101 polymyxin- and carbapenem-resistant K. pneumoniae (PR-CRKP) strains based on single nucleotide polymorphism (SNP) sequences. From left to right: isolation regions and years, sex, age, MIC, ST, KL, carbapenemase genes, Klebsiella species, other β-lactamases and polymyxin-resistance-related genes.

The 101 PR-CRKP strains were isolated from the lower respiratory tract (67.33%, 68, including 48 sputum and 20 bronchoalveolar lavage fluid [BALF] specimens), urine (15.84%, 16), blood (5.94%, 6), wounds (4.95%, 5, including ear, eye, and skin and soft tissue), pleural effusion (1.98%, 2), bone marrow (0.99%, 1), ascites (0.99%, 1), and tissue (0.99%, 1). As shown in Fig. 2 and 3, high-level PRKP (MIC, >16 μg/mL, 58 isolates) were mainly distributed in Beijing (77.50%, 31/40), Hunan (63.89%, 23/36), Shanxi (80.00%, 4/5), and Guangxi (100.00%, 1/1), whereas the low-level ones (MIC, ≤16 μg/mL, 43 isolates) were found mainly in Henan (100.00%, 19/19). No PR-KP isolates were identified from Hebei.

### Antibiotic resistance profiles.

As shown in Table 1, among 101 PR-CRKP strains, more than half of the strains were nonsusceptible to the following antibiotics: aztreonam (90.10%, 91/101), levofloxacin (89.11%, 90/101), ciprofloxacin (88.12%, 89/101), tobramycin (68.67%, 57/101), and amikacin (55.45%, 56/101), followed by trimethoprim-sulfamethoxazole (44.55%, 45/101). Only 14.85% (15/101) were nonsusceptible to tigecycline.

**TABLE 1:** Antimicrobial susceptibility profiles and MIC values of 10 antibiotics of the 101 PR-CRKP^*a*^

Antibiotic	Breakpoint (S, I, R) (μg/mL)	Range (μg/mL)	MIC_50_ (μg/mL)	MIC_90_ (μg/mL)	No. of strains (%)	
					Susceptible	Nonsusceptible^*b*^
IMP	≤1, 2, ≥4	2 to ≥16	≥16	≥16		
MEM	≤1, 2, ≥4	2 to ≥16	≥16	≥16		
PB	NA, ≤2, >2	4 to >256	64	>256		
ATM	≤4, NA, ≥16	4 to ≥64	≥64	≥64	10 (9.90)	91 (90.10)
AK	≤16, NA, ≥64	≤2 to ≥64	≥64	≥64	45 (44.55)	56 (55.45)
TOB	≤4, NA, ≥16	≤1 to ≥16	≥16	≥16	26 (31.33)	57 (68.67)
CIP	<0.25, 0.5, ≥1	≤0.25 to ≥4	≥4	≥4	12 (11.88)	89 (88.12)
LEV	≤0.25, 1, ≥2	≤0.12 to ≥8	≥8	≥8	11 (10.89)	90 (89.11)
TGC	≤2, 4, ≥8	0.25 to 8	1	4	86 (85.15)	15 (14.85)
SXT	≤40, NA, ≥80	≤20 to ≥320	≤20	≥320	56 (55.45)	45 (44.55)

a NA, not available according to CLSI M100; IMP, imipenem; MEM, meropenem; PB, polymyxin B; ATM, aztreonam; AK, amikacin; TOB, tobramycin; CIP, ciprofloxacin; LEV, levofloxacin; TGC, tigecycline; STX, sulfamethoxazole/trimethoprim; S, susceptible; I, intermediate; R, resistant.

b Nonsusceptible contained resistant and intermediary isolates.

### Molecular characteristics of PR-CRKP.

Of 101 PR-CRKP strains, 10 (9.90%) were confirmed as *K. quasipneumoniae* based on WGS. A total of 92 (91.09%) were identified as carbapenemase-producing K. pneumoniae (CPKP) (82.18%, 83/101) and *K. quasipneumoniae* (8.91%, 9/101). Of 10 *K. quasipneumoniae* strains, 9 were isolated from two hospitals in Hunan. Further, of 92 CPKP strains, five carbapenemase types were identified, namely, *bla*_KPC-2_ (66.67%, 68/101), *bla*_NDM-1_ (16.83%, 17/101), *bla*_NDM-5_ (0.99%, 1/101), *bla*_IMP-4_ (4.95%, 5/101), and *bla*_IMP-38_ (0.99%, 1/101). Notably, 2 PR-CRKP (1.98%) strains harbored both *bla*_KPC-2_ and *bla*_NDM-1_.

As shown in Fig. 3, for other β-lactamases, *bla*_SHV_ (89.11%, 90/101) was mostly detected, followed by *bla*_CTX-M_ (60.40%, 61/101, and 44 PR-CRKP strains harbored *bla*_CTX-M-65_) and *bla*_TEM_ (53.47%, 54/101).

A total of 21 individual STs were distinguished, with ST11 dominating (68/101, 67.33%). There was no relationship between ST type and polymyxin resistance level (*P = *0.8159). Furthermore, KL47 accounted all the PR-CRKP for 29.70% (30/101), followed by KL64 (27/101, 26.73%), unclassified (10/101, 9.90%), and KL102 (6/101, 5.94%).

Notably, of 10 *K. quasipneumoniae* strains, 7 ST types were designated, 9 were isolated from Hunan, and 9 harbored metalloenzyme genes, namely, 5 *bla*_NDM-1_ and 4 *bla*_IMP-4_.

### Polymyxin resistance mechanisms.

**FIG 4:**
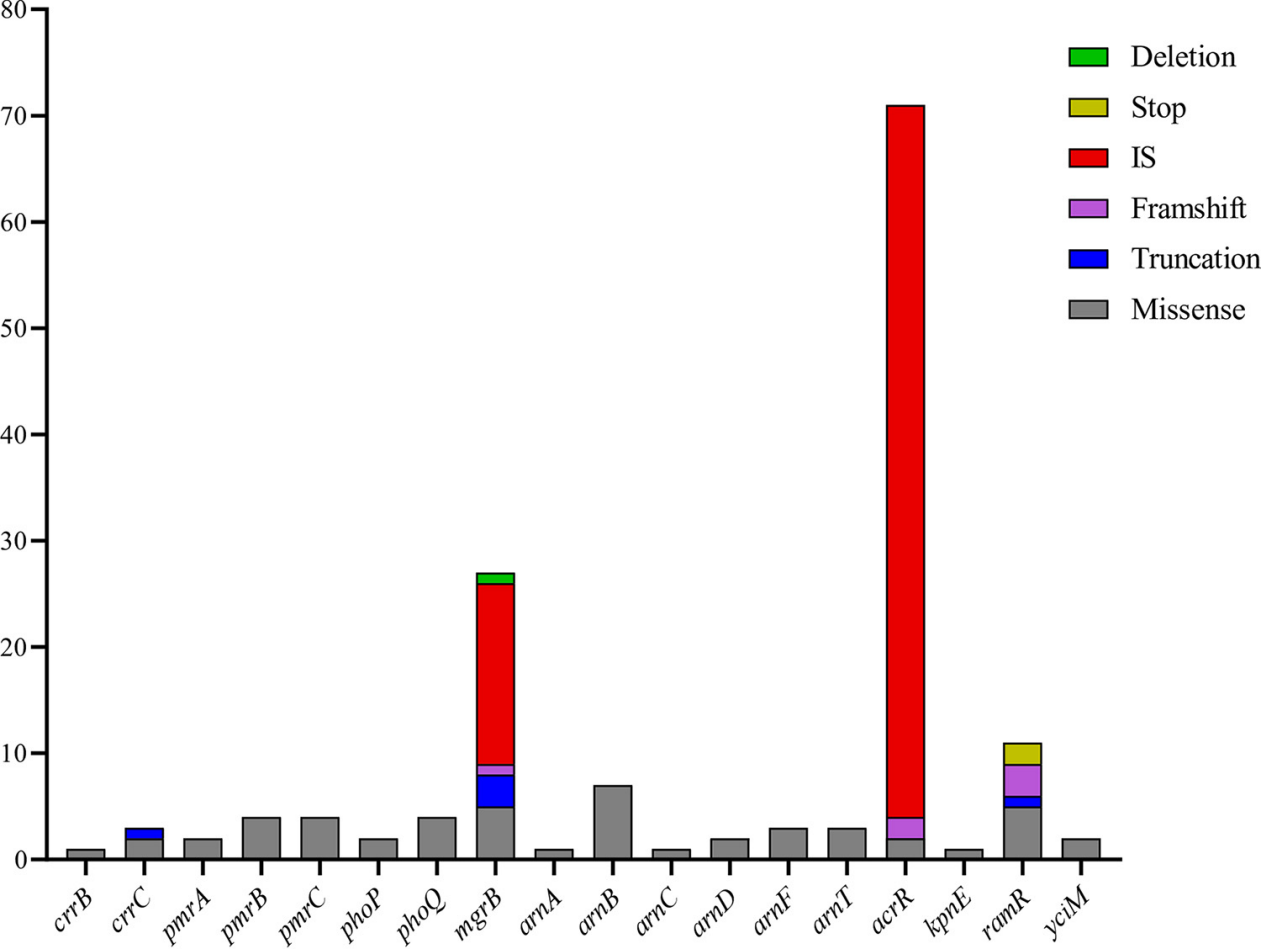
Frequencies of deleterious gene variations conferring polymyxin resistance.

The polymyxin resistance mechanism is shown in Table 2 and Fig. 3 and 4. Of 101 PR-CRKP strains, 168 variations at a variety of sites were identified across all 26 above-mentioned genes possibly involved in polymyxin resistance. The gene variations remaining after removing the neutral variations are listed in Table 2. The *acrR* gene mutation was the most frequently identified (71, 70.29%), followed by *mgrB* (27, 26.73%), *ramR* (11, 10.89%), and *arnB* (7, 6.93%).

**TABLE 2:** Distribution of polymyxin resistance-related gene mutations among 101 polymyxin- and carbapenem-resistant K. pneumoniae isolates^*a*^^,^^*b*^

Gene (*n*_1_)	Gene mutation (+, −/*n*_2_)
*crrA*(0)	V167I (−/1), K228R (−/2), D198N (−/2), deletion (−/43)
*crrB*(1)	1−249 aa del (−/43), C68S (−/20), Q301K (−/2), I27V (−/2), I66V (−/2), Q239H (−/2), T76A (−/2), **V193G (+/1)**, T276A (−/1), Q287K (−/1), M63I (−/1)
*crrC*(2)	1−22 aa del (−/43), A15V (−/4), V84L (−/3), V82I (−/1), **P68T (+/1)**, **107−129 aa del (+/1)**
*pmrA*(2)	A41T (−/4), M66I (−/3), I220N (−/2), D221E (−/2), G53V (+/1), D86E (+/1), E57G (−/1)
*pmrB*(4)	T246A (−/71), **R256G (+/51)**, L213M (−/7), **P344L (+/3)**, G358A (−/3), K220Q (−/3), S363I (−/3), **T157P (+/1)**, M11I (−/1), G345E (−/1)
*pmrC*(4)	I138V (−/92), Q319R (−/81), C27F (−/72), D249E (−/6), L39V (−/3), T224K (−/3), V36I (−/3), **P287S (+/2)**, D75E (−/2), **S257L (+/1)**, **A223S (+/1)**, E408K (−/1), K509T (−/1), I142V (−/1), A162V (−/1)
*pmrD*(0)	T60M (−/8), G80D (−/2)
*phoP*(2)	**D191A (+/2)**, E82D (−/1)
*phoQ*(4)	**P424L (+/3)**, G150D (−/2), **D438E (+/1)**, A145S (−/1), T156S (−/1)
*mgrB*(27)	**C28R (+/1), M1V (+/1), I45F (+/1), T-35C (+/2), deletion (+/1), IS*903* (+/4), IS*kpn14* (+/9), IS*kpn18* (+/2), IS*kpn26* (+/2), disrupt (+/3), 7Vfs (+/1)**
*arnA*(0)	T450N (−/8), L260I (−/5), S18A (−/4), K442N (−/2), A22V (−/1), S061T (−/1), N660H (−/1)
*arnB*(7)	D101A (−/90), T272A (−/5), D274E (−/5), A148V (−/3), **A124T (+/1)**, **R269C (+/1)**, **S371F (+/1)**, **G367V (+/1)**, **P264S (+/2)**, **G36D (+/1)**, I115V (−/1), A261T (−/1), V308A (−/1), I89V (−/1)
*arnC*(1)	T30S (−/2), A36T (−/1), **E204V (+/1)**, I286V (−/1)
*arnD*(2)	L94I (−/20), V300I (−/15), I53V (−/14), **S271C (+/2)**, V187T (−/2), D209E (−/1), V247E (−/1), I300V (−/1), S164P (−/1), S188F (−/1), A225P (−/1)
*arnE*(0)	
*arnF*(3)	A45P (−/8), **P100L (+/3)**, G46S (−/1), G89A (−/1)
*arnT*(3)	H156Q (−/12), I117V (−/11), D441E (−/5), **G164S (+/2)**, S404D (−/2), T153A (−/2), S157R (−/2), K372R (−/2), **S404C (+/1)**, S404S (−/1), K337N (−/1), A153V (−/1), V299M (−/1)
*acrA*(0)	A188T (−/74)
*acrB*(0)	**R716L (−/68)**, E639D (−/4)
*acrR*(71)	**IS*kpn26* (+/67)**, **L34F (+/2), 81Ffs (+/1), 121Gfs (+/1)**
*kpnE*(1)	**G67V (+/12)**, K112Q (−/7), **A55V (+/1)**
*kpnF*(0)	
*ramA*(0)	Deletion (−/10)
*ramR*(11)	**Q122**^*c*^ **(+/2)**, A6V (−/8), **L66del (+/2)**, D77H (−/2), H186N (−/2), V67del (−/2), **R3H (+/1)**, **A159T (+/1)**, **1−38 aa del (+/1)**, **E165K (+/1)**, **G151D (+/1)**, **L111del (+/1)**, **S112R (+/1)**, A20T (−/1), I141T (−/1), A19V (−/1)
*LpxM*(0)	S253G (−/80), **T229S (+/69)**, **N7I (+/32)**, N6K (−/3), E76D (−/1), deletion (−/10)
*yciM*(2)	**N212T (+/83)**, N212S (−/4), A265V (−/3), V163M (−/3), **R350C (+/2)**, A231T (−/1), V298I (−/1)
*soxS*(0)	I26V (−/10), R29K (−/2)
*soxR*(0)	D142E (−/10), E143D (−/2)

a Gene mutations, involving genes deleted, stopped, truncated, frameshifted, or inserted by an insertion sequence.

b n_1_, the number of deleterious mutations of the corresponding genes in the current study; n_2_, the number of strains with a gene variation; +, the mutation was predicted as the deleterious mutation; −, the mutation was predicted as the neutral mutation; fs, frameshift mutation.

c Stop codon. Del, deletion. The bold variation shows the deleterious variation defined in this study.

**FIG 5:**
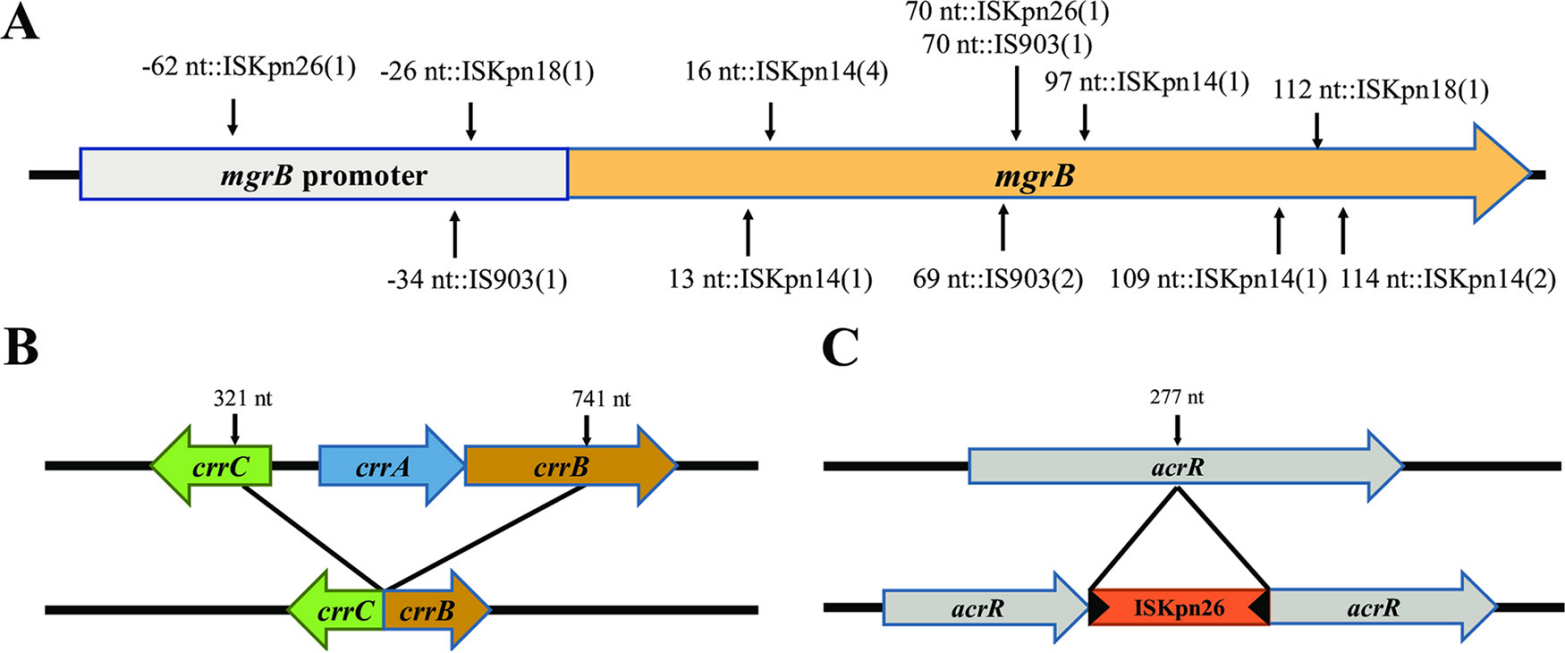
(A to C) Schematic illustrations of (A) insertion sequences (IS; IS*kpn14*, IS*903*, IS*kpn26*, and IS*kpn18*) occurring at different insertion sites of *mgrB*; (B) the structure of *crrCAB* with the splicing mutation occurring at the 321st nt of *crrC* and the 741st nt of *crrB*, resulting in the *crrA* deletion; and (C) the IS (IS*kpn26*) occurring at the 277th nt of *acrR*.

Moreover, the *mgrB* gene, as the most important polymyxin-related gene, was frequently disrupted and inactivated by insertion sequences (IS; 62.96%, 17/27), namely, IS*kpn14* (52.94%, 9/17), IS*903* (23.53%, 4/17), IS*kpn26* (11.76%, 2/17), and IS*kpn18* (11.76%, 2/17), shown in Fig. 5A. No other TCS-related genes were inserted by ISs. Other mutations on *mgrB*, predicted as deleterious, included missense mutations (3 strains, M1V, C28R, and I45F), a frameshift mutation (1, at the 141st site), overall missing (1), and disruptions (3, all truncated to nucleotide [nt] −30), as shown in Fig. 3. The nucleotide substitution (T-35C) at the promoter region of the *mgrB* gene (–10 region) was identified in two strains via BPROM. In addition, all 101 PR-CRKP strains were divided into the *mgrB*-mutation group or not. The Fisher exact test and Mann-Whitney U test showed that the *mgrB* inactivation was significantly relevant with a higher polymyxin resistance level (*P < *0.001).

Missense mutations were observed in other TCS genes, *pmrB* (4 strains), *pmrC* (4 strains) *phoP* (2 strains), and *phoQ* (4 strains). *pmrD* was otherwise relatively conserved and had no deleterious variant. The splicing mutations (42.57%, 43/101) in *crrB* and *crrC* genes were ubiquitous and caused *crrA* deletion and *crrCAB* inactivation; however, this did not increase the MIC of polymyxin. PR-CRKP strains (36.63%, 39/101) harbored no *crrCAB* system (Fig. 3 and 5B). Furthermore, we divided these strains into two groups, harboring a deletion-splicing mutation or not. Fisher’s exact test revealed that *crrCAB* was relevant with ST and KL (both *P < *0.0001). Then the χ^2^ test showed a significant relationship between ST11 and *crrCAB* (*P < *0.001), as well as KL47 and *crrCAB* (*P = *0.005).

The efflux pump *AcrAB*-related genes involved in polymyxin resistance were also evaluated. The IS*kpn26*-inserted *acrR* genes, the repressor of the *AcrAB*, were observed in 67 strains at the same site (+277 nt) (67/101, 66.33%) (Fig. 5C). The PR-CRKP strains were then divided into two groups with or without IS*kpn26*-mediated insertion of the *acrR* gene, and it revealed that ST, especially ST11, was significantly associated with this insertion (*P* < 0.001). Furthermore, one missense mutation (L34F) and two shift mutations (81Ffs, 121Gfs) were also detected in the *acrR* gene. The *ramR* gene, the negative regulator of *ramA*, which can positively regulate efflux pump expression, was the second-most-frequently mutated gene. The amino acid deletions occurred at amino acids 66 and 111 and were overall missing from amino acids 1 to 38. One stop codon was detected at amino acid 122 (Q122*) of the *ramR* gene in 2 strains. One missense mutation was found in *kpnE*, an efflux pump-related multidrug resistance gene. No deleterious mutation was identified among the *acrA*, *acrB*, *kpnF*, *ramA*, *soxS*, and *soxR* genes.

In the genes related to LPS synthesis and modification, the missense mutations were identified in the *arnB* (6 strains) which was one part of *arnBCADTEF* operon and *yciM* (1 strain).

The *mcr* gene was detected only in strain CJFH07 (*mcr-8.2*), in which the missense mutation in *mgrB* and *crrB* and disruption of *acrR* also played a major role in polymyxin resistance (14).

However, the underlying resistance mechanism of 26 PR-CRKP strains (25.49%) remained elusive.

### Complementation experiment and mRNA expression.

**FIG 6:**
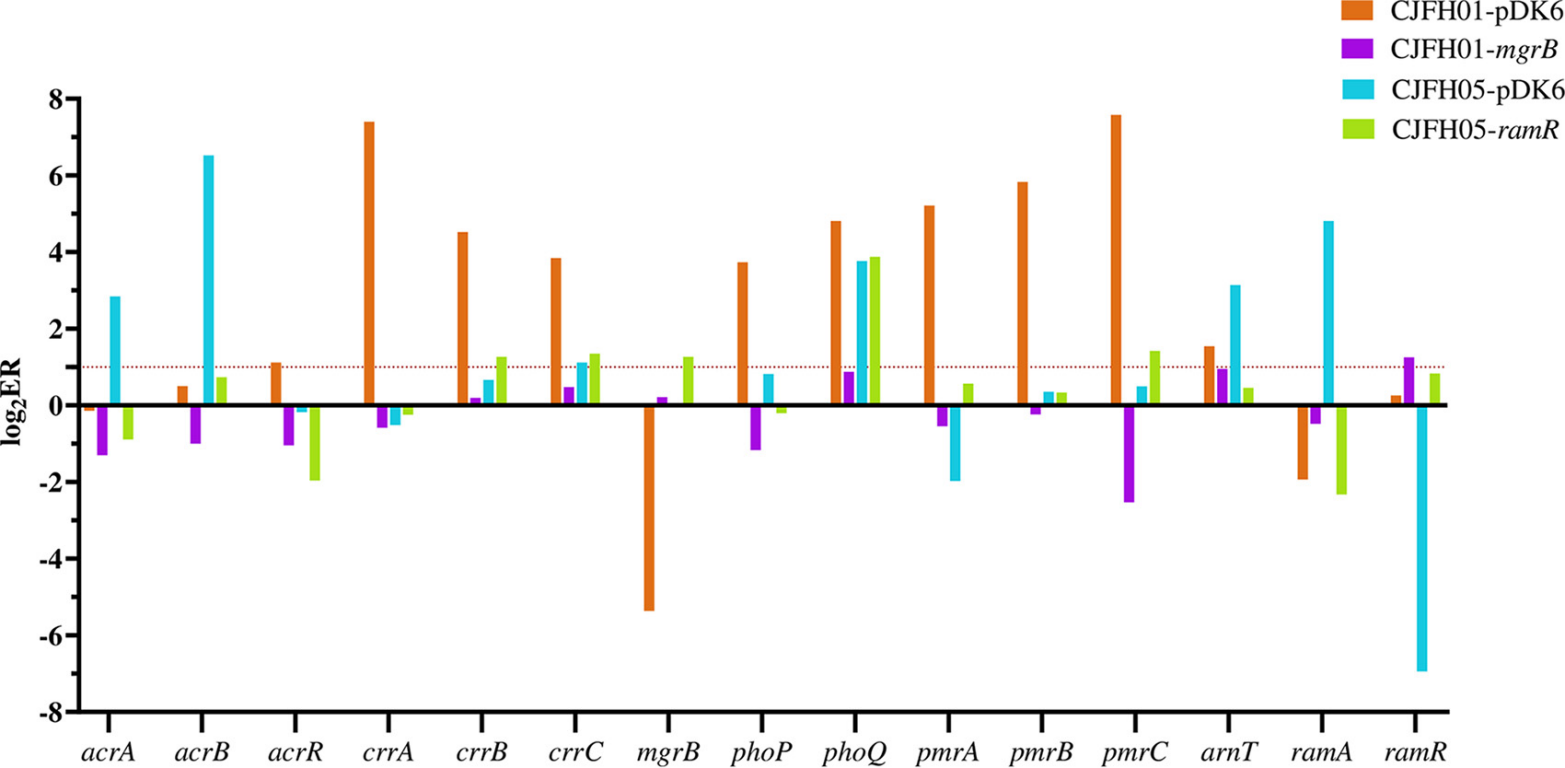
Assessment of mRNA expression. The *x* axis represents the detected genes, including the proven and putative polymyxin resistance gene and the efflux pump-related gene. The *y* axis represents the logarithm of relative expression (RE). RE is the expression ratio (ER) of detected strains divided by the ER of ATCC 13883. ER is the average of the ratio of the detected genes to the *rpoB* gene for each strain.

The complementation experiment of the *ramR* and *mgrB* genes was performed for strains CJFH05 and CJFH01, whose resistance only resulted from the mutation of *ramR* and the insertion of the *mgrB* promoter region, respectively. The polymyxin MIC of CJFH05 fell from 8 μg/mL to 1 μg/mL, whereas the MIC of CJFH01 decreased from 256 μg/mL to 0.5 μg/mL after the complementation experiment with the *ramR* and *mgrB* promoter, while the MICs of the control strains were the same as those of the original strains. The mRNA assessment is shown in Fig. 6. The expression of the polymyxin resistance genes (*crrA/B/C*, *pmrA/B/C*, and *arnT*) and efflux pump genes (*acrA/B* and *ramA*) reduced to the same level as ATCC 13883 after the plasmid was complemented, suggesting that the mutations in *ramR* or *mgrB* played a critical part in polymyxin resistance.

## DISCUSSION

Polymyxin has become the antibiotic of last resort against the challenge of CRKP infections (15). However, K. pneumoniae isolates that are resistant to both polymyxin and carbapenem have been documented, and the underlying mechanism was under surveillance (10, 16, 17). Predominant molecular characteristics of PR-CRKP strains may differ geographically and over time (10, 16, 18, 19). Therefore, in the study, the genomic survey of clinical PR-CRKP was conducted to investigate the genetic alterations in these isolates circulating in China.

Most PR-CRKP strains were recovered from the lower respiratory tract, similar to a previous survey in China (20). Furthermore, a considerable number of PR-CRKP strains were isolated from children (22.77%) and elderly patients (41.58%).

*K. quasipneumoniae*, as a novel Klebsiella sp., was commonly misidentified as K. pneumoniae in the clinical laboratory (21). WGS can be used to distinguish the species, and its distribution may be related to the regions and infection sites (13). However, many reports did not distinguish *K. quasipneumoniae* from K. pneumoniae, and few reports of polymyxin- and carbapenem-resistant *K. quasipneumoniae* were available online (3, 22, 23). In this study, about 10% of K. pneumoniae were reidentified as *K. quasipneumoniae*, similar to a previous report from Japan (9.2%) and higher than in Switzerland (4.59%), India (5.23%), and Nigeria (3.73%) (24–27).

Phylogenomic comparison revealed that this national collection of PR-CRKP showed diverse characteristics, with the presence of epidemic clones. Unlike in European countries, where ST258 was prevalent (28), the ST11 *bla*_KPC-2_-positive CRKP predominated in China (29). In our study, ST11 accounted for 66.67% of all PR-CRKP strains. The KPC-2-producing strains predominated in our PR-CRKP strains, and the IMP-producing K. pneumoniae strains were clustered in only Hunan, similar to previous reports (1, 30). Coexistence of dual carbapenemases (*bla*_NDM-5_ and *bla*_KPC-2_) in 2 strains was identified.

For other β-lactamases, *bla*_SHV_ (89.11%) was the most frequently detected β-lactamase among 101 PR-CRKP strains, while chromosome-harbored *bla*_SHV_ and *bla*_OKP_ were intrinsically carried genes were demonstrated earlier (31). The chromosomal *bla*_OKP_ family, a phylogenetic marker found in group KpII, shared the same ancestry as *bla*_SHV_ and *bla*_LEN_ (32, 33). A total of 60.40% (61/101) of PR-CRKP strains harbored *bla*_CTX-M_, in which *bla*_CTX-M-65_ predominated (44/61, 72.1%). By comparison, *bla*_CTX-M-15_ was often documented as the predominant *bla*_CTX-M_ β-lactamases (34, 35), and this might be explained by geographic differences.

Mutations in *mgrB* and TCS-related genes were notable in the current study. *mgrB* gene inactivation was the underlying mechanism of polymyxin resistance of K. pneumoniae reported worldwide (4, 5, 36, 37), as confirmed in our study (26.73%, 27/101). IS-medicated insertion was the main reason for *mgrB* inactivation (65.38% in this study). Furthermore, the IS types involved IS*kpn14* (52.94%), followed by IS*903* (23.53%,), IS*kpn26* (11.76%), and IS*kpn18* (11.76%), consistent with previous reports (16, 18, 38, 39). IS*kpn14* was prevalent as the main *mgrB*-inserted type in our study, which might vary geographically, such as IS*5*-like in Italy and Switzerland (4, 37) and IS*kpn26* in Taiwan (16). The *mgrB* insertion by IS*kpn18* was reported first in 2018 in a Chinese study and rarely elsewhere (40). Furthermore, the ISs were inserted in certain hot spots as previously reported, such as +69 to ~+75 nt (4, 5, 16, 18, 37), as also observed in the present study.

The dysfunction of the *crrCAB* system might mediate the polymyxin resistance (41, 42). In the present study, if the whole *crrCAB* system was deleted, as observed in almost all ST11 K. pneumoniae strains, the polymyxin MIC remained unchanged. The regulatory pathway by which *crrAB* regulated polymyxin resistance was only via *crrC* (41, 43). Consequently, *crrCAB* cannot contribute to polymyxin resistance if the whole system is removed. However, the splicing mutations of *crrB* and *crrC* may lead to the loss of the *crrCAB* function. The role of the *crrCAB* system in the polymyxin resistance of K. pneumoniae requires further in-depth study. In addition, previously unreported novel mutations were identified in our study; *crrB* (V193G), *crrC* (P68T), and *crrC* (107- to 129-amino acid [aa] deletion) were reported.

As for other TCS-related genes, the mutation in *pmrB* (R256G) was identified in our study, but its role in polymyxin resistance was controversial. It was considered a deleterious mutation according to the PROVEAN result (44). However, Sampaio reported that the substitutions in *pmrB* (T246A and R256G) were not involved in polymyxin resistance (45). Campos also documented that the expression of the *pmrAB* system with the above-described mutations had no significant influence on polymyxin resistance (15). Therefore, in our study, we considered it a nondeleterious mutation.

The inactivation in the repressors of efflux pump *AcrAB* genes has been documented to contribute to polymyxin resistance (10, 11). The efflux function of *AcrAB* would be hyperactive if *acrR*, the repressor of the *AcrAB*, was inactivated (11). In the present study, the inactivated *acrR* genes inserted coincidently at +277 nt by IS*kpn26* were present in about 65.69% of PR-CRKP strains, and all of them belonged to ST11. A total of 25.74% (26/101) strains only harbored this *acrR* gene mutation and showed low-level polymyxin resistance. Similar to our results, Yang et al. demonstrated that IS*kpn26* was always inserted into the *acrR* gene, resulting in *acrR* inactivation and then *acrAB* gene overexpression and multidrug resistance, and the insertion was mainly relevant to the ST11-KPC-2-producing CRKP (46). In their study, several K. pneumoniae isolates presenting an IS*Kpn26* interrupted the *acrR* gene and caused only a limited alteration of colistin MIC after exposure with 1-(1-naphthylmethyl)-piperazine (NMP), which was an efflux pump inhibitor. It seemed that the truncated *acrR* played only a small part in polymyxin resistance. Taken together, ST11 CRKP, as the predominate ST type in China, should be highlighted due to the overactivated efflux pump mediated by the IS*kpn26*-inserted *acrR* genes. However, the underlying mechanism remains elusive, and further study should be performed.

The *ramR* gene might repress the expression of *ramA*, which can regulate other efflux pumps, such as *AcrAB*, and then LPS modification and eventually mediate the decreased susceptibility to polymyxin in K. pneumoniae (47, 48), while in our study, the complementation experiment of the *ramR* gene was employed, indicating that the mutation of *ramR* will result in polymyxin resistance. Molecular epidemical studies of the *ramR* gene in PR-CRKP have rarely been reported, and the present study showed that mutations in the *ramR* gene were diverse, containing deletion, missense mutation, nonsense mutation, and truncation. This deserves more attention.

The *arnBCADTEF* operon, *lpxM* and *yciM*, mediated LPS biosynthesis and modification (3, 15, 22). In the deleterious mutations in the above-mentioned genes, the mutations in *arnB* were prevalent, similar to the results of a previous study (14). However, in the present study, though predicted as deleterious mutations by PROVEAN, the genes *lpxM* (T229S), *lpxM* (N7I), and *yciM* (N212T) were 100% similar to the reference sequences in GenBank (*yciM* [N212T]: NCBI reference sequence WP_002901781.1; *lpxM* [T229S] and *lpxM* [N7I]: NCBI reference sequence: WP_072353980.1) after alignment using the NCBI BLASTp online tool. Consequently, we defined them as nondeleterious variations. Multiple new mutations were identified in various genes in this study, but the significance of these mutations and their contribution to polymyxin resistance remained unclear.

In the current study, only one isolate carried the *mcr* gene, showing that polymyxin resistance was most associated with chromosome variation. However, the polymyxin resistance mechanisms of the 26 isolates remained unclear, indicating the presence of an alternative mechanism regulating the expression of these genes, which prompts the demand for exploration of polymyxin resistance.

Our study was limited by several factors. First, the majority of the isolates were collected from a few provinces/cities in China. Therefore, the national surveillance program should be further developed. Second, the underlying resistance mechanism of 26 PR-CRKP strains remained unclear, and we need to investigate this further.

In summary, the current study demonstrated the genetic diversity of the underlying polymyxin resistance mechanism of 101 PR-CRKP strains collected in China. Polymyxin resistance in the study is multifaceted and attributed to mutations in chromosomal genes leading to lipopolysaccharide modification or efflux of polymyxin through pumps. With few transmissible *mcr* genes identified, the prevalence of polymyxin-resistant strains was low in this unit. Furthermore, the high IS-inserted *mgrB* inactivation, the close relationship between ST11, the deletion or splicing mutations of *crrCAB*, and specific features of PR-*K. quasipneumoniae* constituted notable features of our PR-CRKP strains. The current study will help us to understand the diversity of polymyxin resistance in PR-CRKP circulating in China.

## MATERIALS AND METHODS

### Ethics approval.

The use of the K. pneumoniae isolates for research purposes was approved by the ethics committee of the China-Japan Friendship Hospital (2022-KY-054). The written informed consent from participants was exempted, and the privacy of involved subjects was not affected.

### Strain collection and antibiotic sensitivity test (AST).

A total of 662 unduplicated CRKP strains, confirmed as K. pneumoniae by MALDI-TOF MS (Bruker Daltonics, Germany), were collected from 8 hospitals in 6 provinces/cities across China. Their MIC of carbapenems, polymyxin, and tigecycline were determined by BMD. A MIC of polymyxin of >2 μg/mL was defined as resistance recommended by CLSI M100 guidelines (49), a MIC of tigecycline of >2 μg/mL was defined as nonsusceptible according to the FDA (https://www.fda.gov/). The MIC of other antibiotics, including amikacin, aztreonam, levofloxacin, ciprofloxacin, tobramycin, and sulfamethoxazole/trimethoprim, were determined via the Vitek-2 system with N335 susceptibility cards (bioMérieux, France).

### Epidemiological and clinical data.

Medical reports were accessed to collect demographic variables for all patients; 46 (6.95%) non-PR-CRKP cases were lost for follow-up, and their data were unavailable. The following data were retrospectively collected: gender, age, and place of residence.

### Definition.

The strains with a MIC of >16 μg/mL were defined as having high-level polymyxin resistance as previously described (50). Deleterious mutations were defined as the mutations of certain genes predicted to affect protein function using PROVEAN.

### WGS.

The whole genomes of PR-CRKP strains were sequenced using an Illumina NovaSeq PE150 at the Beijing Novogene Bioinformatics Technology Co., Ltd. Raw reads were filtered to remove low-quality sequences and adaptors. *De novo* assembly was conducted using SOAP *de novo* 2.04, SPAdes 3.10.0, and ABySS 1.3.7. The assembly results were integrated with CISA 1.3 software. The gap in preliminary assembly results was filled using Gapcloser 1.12.

### Bioinformatics analysis.

Genotyping containing species reidentification, multilocus sequence typing (MLST), and identification of antimicrobial resistance genes, including carbapenemase and other β-lactamases, was conducted using Kleborate 2.0.4 (https://github.com/katholt/Kleborate). Capsular locus typing (KL-typing) was performed with the Kaptive webtool (https://kaptive-web.erc.monash.edu/).

### Identification of mutations in proven or putative polymyxin resistance-related genes in K. pneumoniae.

The mutations in the *mgrB* gene and its promoter region were identified using NCBI-blast+ 2.11.0 with K. pneumoniae MGH 78578 (GenBank accession number CP000647) and *K. quasipneumoniae* strain ATCC 700603 (GenBank accession number CP014696) as reference strains.

The amino acid mutations encoded by proven or putative polymyxin resistance-related genes, such as TCS-related genes (*pmrA*, *pmrB*, *pmrC*, *pmrD*, *phoP*, *phoQ*, *crrA*, *crrB*, *crrC*), *arnBCADTEF* operons *arnB* and *yciM*, resistance-nodulation-division (RND) multidrug efflux pump *AcrAB*-involved and -regulating genes (*acrA*, *acrB*, *acrR*, *ramA*, *ramR*, *soxS*, and *soxR*), small multidrug resistance family (SMR) efflux pump-related genes (*kpnE*/*kpnF*), etc., were defined by alignment with the MGH 78578 and ATCC 700603 genomes through blast+ 2.11.0 with the E value set at 1e-50. The ISfinder online tool (https://isfinder.biotoul.fr/blast.php) was used to identify the insertion sequence (IS) type. The PROVEAN platform (http://provean.jcvi.org/index.php) was used to predict alterations in the biological functions of the above-described proteins. The promoter region was predicted using BPROM (http://linux1.softberry.com/berry.phtml).

### Phylogenetic reconstruction.

For the phylogenetic analysis, high-quality single-nucleotide polymorphisms (SNPs) were obtained using snippy 3.2-dev against MGH 78578. The alignment file was filtered from variants with elevated densities of base substitutions as a putative recombination event using Gubbins 2.4.1. Then snp-sites 2.5.1 was used to reduce the filtered alignment to the core polymorphic sites. Next, the core alignment output was used to create a randomized accelerated maximum likelihood (RAxML) tree with RAxML 8.2.12 under the general time reversible (GTR)-Gamma model. The tree layout was graphically edited using iTOL 5.6.

### Statistical analysis.

The differences between the ST type and *crrCAB*, the KL type and *crrCAB*, and the resistance level and related genes were evaluated via the χ^2^ test or Fisher’s exact test for categorical variables and the Mann-Whitney U test or continuous variables, as appropriate, using the IBM SPSS Statistics software packages 22.0 (SPSS, Inc., Chicago, IL). A *P* value of <0.05 was considered statistically significant.

### Complementation experiment.

Apramycin-resistant pDK6 was used as the vector as previously described, but with modifications (51). The *ramR* and *mgrB* sequences were amplified by PCR via PrimeSTAR Max DNA polymerase (TaKaRa, Dalian) with primers ramR-F/R (forward [F]: AGCCAAGCTTGCATGCCTGCAGCCGGGTTCGATACTGCGATAA, reverse [R]: ACAGGAAACAGAATTCGAGCTCCGGCATTAAGAACAAACGGCA) and mgrB-F/R (F: AGCCAAGCTTGCATGCCTGCAGAGCCAGCGATGCCAGATTTA, R: ACAGGAAACAGAATTCGAGCTCCGCCAATCCATAAGATAGCCAC). The plasmid was linearized and PCR products were digested using the double-digestion method. Then, T4 DNA ligase (NEB, USA) was selected for ligating the vector at 16°C overnight, and the plasmid was transformed into CJFH05 and CJFH01 and then inoculated on 30 μg/mL apramycin-containing LB ager. The MICs of polymyxin were determined. The control strains were CJFH05 and CJFH01 with empty vector pDK6 transformed named CJFH01-pDK6 and CJFH05-pDK6, respectively.

### mRNA extraction and expression assessment using digital droplet PCR (ddPCR).

Total RNA was extracted from strains CJFH01-pDK6, CJFH01-*mgrB*, CJFH05-pDK6, and CJFH05-*ramR* using the RNeasy minikit (Qiagen) according to the manufacturer’s instructions and reverse-transcribed into cDNA via a RevertAid first-strand cDNA synthesis kit with DNase I (Thermo Fisher Scientific, USA). The ddPCR was performed following the manufacturer’s protocol using QX200 ddPCR EvaGreen supermix (Bio-Rad, USA), a QX200 droplet generator (Bio-Rad), a QX200 droplet reader (Bio-Rad), and QuantaSoft software (Bio-Rad). The *rpoB* gene was used as the internal control. *K. pneumonia* ATCC 13883 was selected as the control strain. The primers used refer to previous studies (14). The relative expression (RE) of each strain was calculated as follows:

$$\text{Expression ratio }\left(\text{ER}\right)=\text{ AVE }\left({\text{copy numbers}}_{\text{detecting gene}}/{\text{copy numbers}}_{\mathrm{rpoB}}\right)$$

$$\text{RE}={\text{ER}}_{\text{testing strains}}/{\text{ER}}_{\text{ATCC 13883}}$$

ER is the average of the ratio of the detecting gene to the *rpoB* gene for each strain. The RE is the ER of detecting strains divided by the ER of ATCC 13883.

### Data availability.

All genome sequences have been submitted to the National Center for Biotechnology Information (NCBI) database with BioProject accession number PRJNA839599 and BioSample accession numbers SAMN28546397 to SAMN28546497. The original data presented in the study are included in the article. Further inquiries can be directed to the corresponding author.
